# Genome-wide identification of *Saccharomyces cerevisiae *genes required for tolerance to acetic acid

**DOI:** 10.1186/1475-2859-9-79

**Published:** 2010-10-25

**Authors:** Nuno P Mira, Margarida Palma, Joana F Guerreiro, Isabel Sá-Correia

**Affiliations:** 1 Institute for Biotechnology and Bioengineering, Centre for Biological and Chemical Engineering, Instituto Superior Técnico, Technical University of Lisbon, 1049-001 Lisboa, Portugal

## Abstract

**Background:**

Acetic acid is a byproduct of *Saccharomyces cerevisiae *alcoholic fermentation. Together with high concentrations of ethanol and other toxic metabolites, acetic acid may contribute to fermentation arrest and reduced ethanol productivity. This weak acid is also a present in lignocellulosic hydrolysates, a highly interesting non-feedstock substrate in industrial biotechnology. Therefore, the better understanding of the molecular mechanisms underlying *S. cerevisiae *tolerance to acetic acid is essential for the rational selection of optimal fermentation conditions and the engineering of more robust industrial strains to be used in processes in which yeast is explored as cell factory.

**Results:**

The yeast genes conferring protection against acetic acid were identified in this study at a genome-wide scale, based on the screening of the EUROSCARF haploid mutant collection for susceptibility phenotypes to this weak acid (concentrations in the range 70-110 mM, at pH 4.5). Approximately 650 determinants of tolerance to acetic acid were identified. Clustering of these acetic acid-resistance genes based on their biological function indicated an enrichment of genes involved in transcription, internal pH homeostasis, carbohydrate metabolism, cell wall assembly, biogenesis of mitochondria, ribosome and vacuole, and in the sensing, signalling and uptake of various nutrients in particular iron, potassium, glucose and amino acids. A correlation between increased resistance to acetic acid and the level of potassium in the growth medium was found. The activation of the Snf1p signalling pathway, involved in yeast response to glucose starvation, is demonstrated to occur in response to acetic acid stress but no evidence was obtained supporting the acetic acid-induced inhibition of glucose uptake.

**Conclusions:**

Approximately 490 of the 650 determinants of tolerance to acetic acid identified in this work are implicated, for the first time, in tolerance to this weak acid. These are novel candidate genes for genetic engineering to obtain more robust yeast strains against acetic acid toxicity. Among these genes there are number of transcription factors that are documented regulators of a large percentage of the genes found to exert protection against acetic acid thus being considered interesting targets for subsequent genetic engineering. The increase of potassium concentration in the growth medium was found to improve the expression of maximal tolerance to acetic acid, consistent with the idea that the adequate manipulation of nutrient concentration of industrial growth medium can be an interesting strategy to surpass the deleterious effects of this weak acid in yeast cells.

## Background

Acetic acid is a byproduct of the alcoholic fermentation carried out by *Saccharomyces cerevisiae*. In the absence of glucose or of any other repressive carbon source, acetic acid can be used as a carbon source by this yeast species through the activity of the anaplerotic glyoxylate cycle and neoglucogenesis [1]. In the presence of glucose, however, these metabolic pathways are repressed leading to the accumulation of acetic acid in the growth medium [1]. During alcoholic fermentation, the concentration of acetic acid can achieve levels that, together with the concentrations of ethanol and other toxic metabolites produced, may lead to fermentation arrest and reduced ethanol volumetric production [2-4]. Acetic acid can also be produced by contaminating lactic and/or acetic acid bacteria in vinification [5]. Acetic acid is also one of the inhibitors of the microbial fermentation of lignocellulosic hydrolysates, a potential substrate for the production of bioethanol and other chemicals (e.g. lactic acid and xylitol) [6-9].

The development of efficient biomass fermentation processes is considered a crucial step to reduce the world's oil demand. Lignocellulosic materials are considered an important alternative for the production of bioethanol which is currently produced from agricultural products [6]. Due to the release of the acetyl groups present in lignin, acetic acid is produced and it will be present in the biomass hydrolysates used for the fermentation step [6]. Other microbial inhibitors are produced during this biomass pre-treatment (e.g. furfural and hydromethylfurfural), although their presence in the hydrolysates can be avoided by process improvement of the plant polymer breakdown [8]. Since lignin is a heavily acetylated polymer, acetic acid will always be present in the final lignocellulosic hydrolysates [6]. Acetic acid is also widely used as a food preservative but the resistance of spoilage yeasts to this weak acid limits its action, with consequent major economic loses in the Food industries [10]. A better understanding of the molecular mechanisms underlying yeast tolerance to acetic acid is urgently needed for the development of yeast-based industrial biotechnology, in particular, from lignocellulosic feedstocks.

In a growth medium with a pH equal or below its p*Ka *(4.7), the undissociated form of acetic acid (RCOOH) prevails. This undissociated form enters the yeast cells by simple diffusion through the plasma membrane lipid bilayer and dissociates in the near-neutral cytosol leading to the accumulation of protons and acetate in the cell interior [11]. The acetic acid-induced intracellular acidification inhibits cell metabolic activity [12,13] and contributes to the dissipation of plasma membrane electrochemical gradient, as proposed to occur under stress imposed by other weak acids [11,14,15]. The recovery of intracellular pH to more physiological values in acetic acid-challenged cells requires the stimulation of the activity of plasma membrane H^+^-ATPase (PM-H^+^-ATPase) Pma1p, which couples ATP hydrolysis to proton extrusion [16]. The expression of the plasma membrane multidrug resistance transporters of the major facilitator superfamily Tpo2p, Tpo3p, Aqr1p and Azr1p, confers resistance to acetic acid and they are thought to mediate the active expulsion of acetate [17-19]. *TPO2*, *TPO3 *and *AQR1 *genes are transcriptionally activated in response to acetic acid [19,20] under the dependence of the transcriptional activator Haa1p [20], which is also a determinant of yeast resistance to acetic acid [19,20]. Haa1p was found to be the main player in the control of yeast genomic expression program in response to acetic acid, being required for the transcriptional regulation of approximately 80% of the acetic acid-responsive genes [20]. Other genes of the Haa1p-regulon are also required for maximal yeast tolerance to acetic acid but the strongest protective effect was registered for the *SAP30 *gene, encoding a subunit of the Rpd3L histone deacetylase complex recently implicated in the regulation of transcriptional response to environmental stress [21]; and for the *HRK1 *gene, whose product is a protein kinase belonging to a family of kinases dedicated to the regulation of plasma membrane transporters [22]. Based on the higher accumulation of acetic acid registered inside acetic acid-challenged Δ*hrk1 *and Δ*haa1 *cells [19,20], it was hypothesized that the role of the Haa1p-mediated signaling pathway in cell protection against acetic acid involves the reduction of intracellular acetate concentration [20].

The aim of this study was to systematically identify, at a genomic scale, the genes required for maximal tolerance to acetic acid in *S. cerevisiae *by screening the EUROSCARF haploid mutant collection (http://web.uni-frankfurt.de/fb15/mikro/euroscarf/) for susceptibility phenotypes towards this weak acid. Three concentrations of acetic acid (70, 90 and 110 mM, at pH 4.5) were tested and approximately 650 genes were identified as resistance determinants. Approximately 75% of these genes are here implicated in yeast tolerance to this weak acid for the first time.

## Results

### Genome-wide identification of determinants of resistance to acetic acid

**Figure 1:**
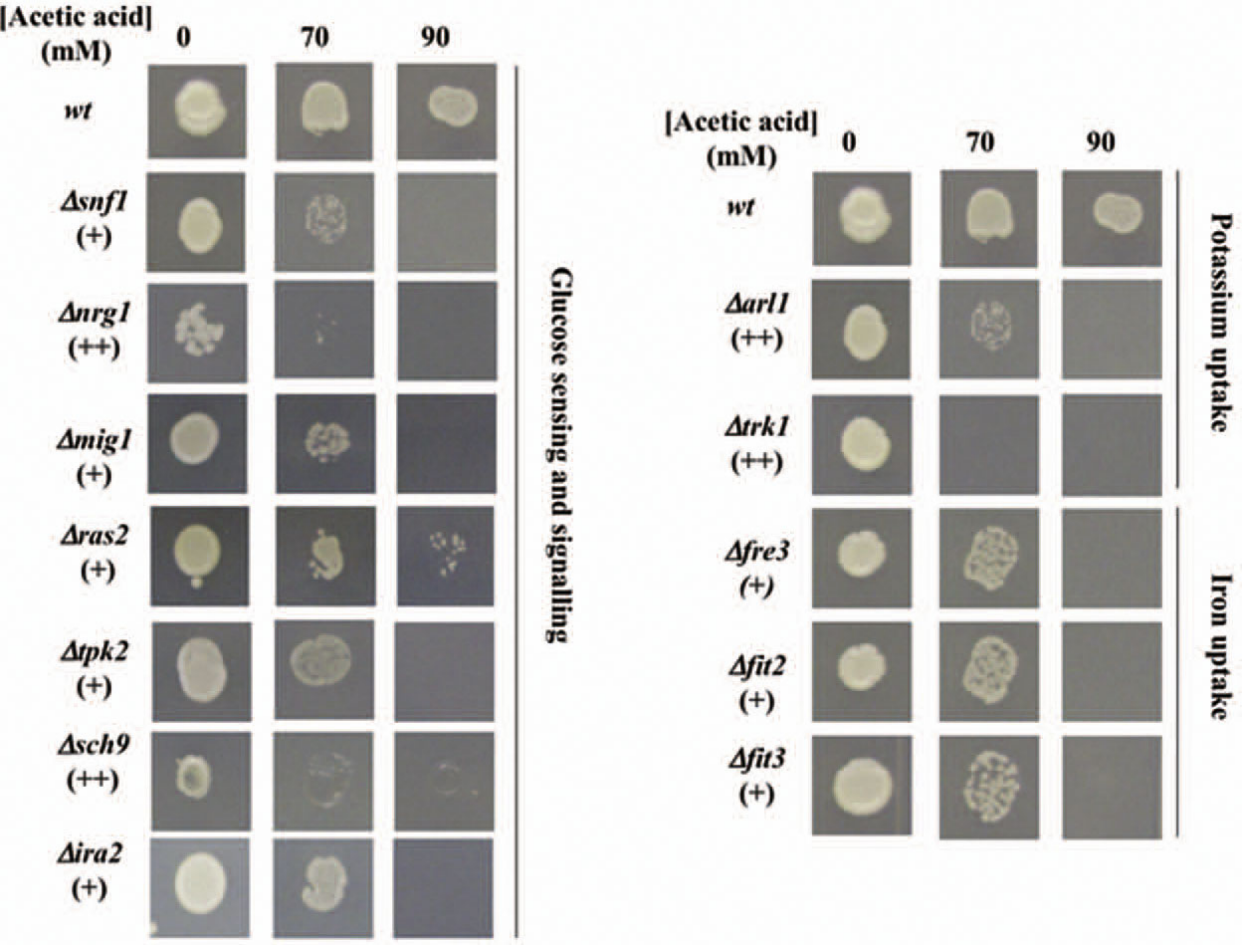
**Comparison of the susceptibility to acetic acid of a set of deletion mutants tested during the yeast disruptome screening**. Cell suspensions of the parental strain BY4741 and of the indicated deletion mutants were cultivated until mid-exponential phase in MM4 growth medium (at pH 4.5) and then inoculated in plates of this same basal growth medium either or not supplemented with acetic acid (70 and 90 mM; at pH 4.5), as described in materials and methods.

**Figure 2:**
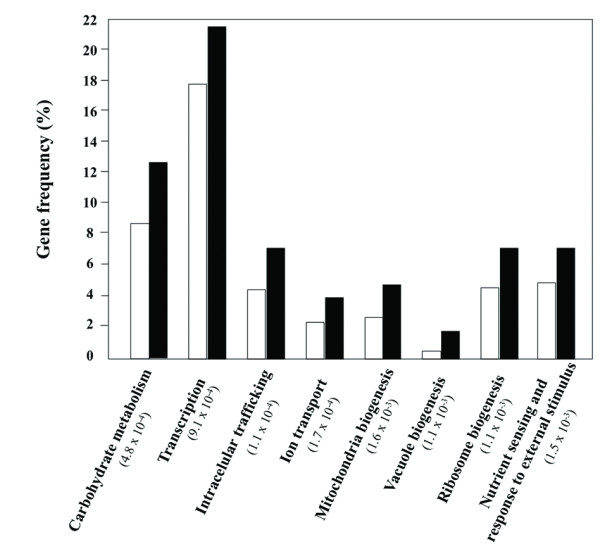
**Clustering, based on physiological function, of yeast genes required for maximal tolerance to acetic acid**. The genes whose elimination was found to increase the susceptibility of *S. cerevisiae *BY4741 to acetic acid, according to the screening of yeast disruptome, were clustered using the MIPS functional catalogue and the enriched functional classes (those having a *p*-value below 0.01) were selected. The frequency in our dataset of acetic acid-tolerance genes (black bars), compared with the frequency of genes obtained when the whole yeast genome is used as an input (white bars), is also indicated.

The chemical genomics analysis performed to identify the genes implicated in *S. cerevisiae *resistance to acetic acid was based on the comparison of the susceptibility to acetic acid (70, 90 and 110 mM, at pH 4.5) of the mutants of the EUROSCARF haploid collection (approximately 5100 mutants individually deleted for non-essential genes) with the parental strain BY4741. Six hundred and forty eight mutants were found to be more susceptible to acetic acid than the parental strain, this corresponding to approximately 13% of the mutant strains tested. However, no resistance phenotypes were registered for any of the three concentrations of acetic acid tested. A full list of the genes whose deletion increased yeast susceptibility to acetic acid is available in Additional file 1, Table S1. Two levels of susceptibility were considered, based on increasing levels of growth deficiency in the presence of acetic acid of the deletion mutants tested, compared to the parental strain, as illustrated in Figure 1. The results obtained for a number of other selected mutants are also available in Additional file 2, Figure S1. The number of determinants of resistance to acetic acid identified in our study is well above the number reported in a previous screening (648 compared to 250) [23], with approximately 150 genes being common to the two datasets. The differences found in the two studies probably result from the different experimental conditions used, specifically: *i) *higher concentrations of acetic acid tested in our study (70-110 mM at pH 4.5 compared to 66.7 mM); *ii) *the use in our study of a minimal growth medium instead of the rich YPD medium; *iii) *the use in our study of cells in mid-exponential phase instead of stationary-phase cells which are more stress resistant [24]. Clustering of the genes required for maximal tolerance to acetic acid, based on their biological function, was performed according to the MIPS functional catalogue (http://mips.helmholtz-muenchen.de/proj/funcatDB/). The frequency of each functional class was compared in our dataset and in the genome and a statistical test was applied to correct the data. The enriched functional classes (those having an associated *p*-value below 0.01) within our dataset of determinants of resistance to acetic acid were: "Ion transport", "Carbohydrate metabolism", "Transcription", "Intracellular trafficking", "Vacuole biogenesis", "Mitochondria biogenesis", "Ribosome biogenesis" and "Nutrient sensing and response to external stimulus" (Figure 2). The "Intracellular trafficking" class is essentially composed by vacuolar sorting proteins (e.g. *VPS1*, *VPS8*, *VPS29*) and proteins belonging to the Multivesicular Body Pathway (*STP22*, *PEP8*, *SNF7*, *VPS36*, etc) whereas the "Transcription" class contains a vast number of genes involved in general transcription activities and in chromatin remodelling. These biological functions are among those required for multidrug resistance in yeast [25] and, thus, their involvement in acetic acid tolerance was expected. The beneficial effect of the expression of genes related to ribosome biogenesis in tolerance to this weak acid is in agreement with the dramatic increase of the degradation rate of ribosomal RNA in acetic acid-stressed cells [26]. The biological role of the genes included in the other enriched functional classes is discussed in the following sections.

#### Genes related to proton homeostasis and to potassium and iron uptake are required for yeast tolerance to acetic acid

**Figure 3:**
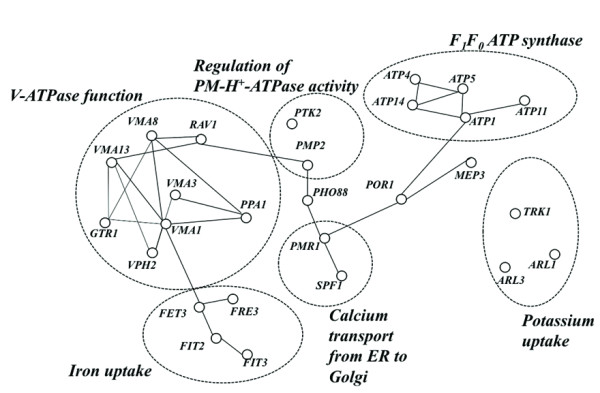
**Interaction network map of the yeast genes required for tolerance to acetic acid that were clustered in the Ion transport functional class**. The genes exerting protection against acetic acid that were clustered in the "Ion transport" functional class were selected and used as an input dataset for the analysis of interactions, at a protein or gene level, using BIOGRID. In the depicted map an interaction is represented by a connection between two nodes.

The "Ion transport" class includes a number of genes related to proton homeostasis, more specifically, to the assembly and/or regulation of the activity of plasma membrane H^+^-ATPase (PM-H^+^-ATPase), of vacuolar H^+^-ATPase (V-ATPase) and of mitochondrial F_1_F_0 _ATP synthase (Figure 3). The coordinate activation of PM-ATPase and V-ATPase is required to counteract intracellular acidification induced by weak acids [14,27-29]. Indeed, the activation of PM-ATPase Pma1p activity was registered in acetic acid-stressed cells and the pH of the vacuolar lumen was found to decrease in these cells, accompanying the decrease of cytosolic pH [16]. By sequestering the exceeding protons present in the cytosol to the vacuole lumen of weak acid-challenged cells, vacuolar acidification may help in the recovery of cytosolic pH to more physiological values [16,28,29].

**Figure 4:**
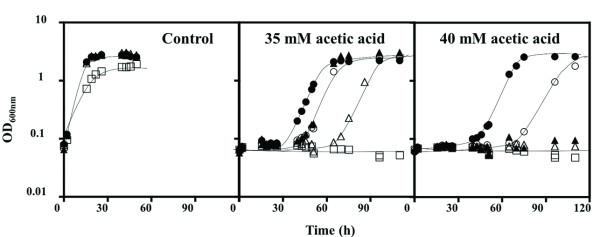
**Effect of the growth medium K^+ ^concentration in the in the acid-induced inhibition of yeast growth**. The susceptibility of yeast cells to acetic acid (35 and 40 mM; at pH 4.0) was compared in an ammonium-derived mineral growth medium supplemented with 1 mM (white squares), 2.5 mM (white triangles), 5 mM (white inverted triangles), 10 mM (white circles) and 20 mM (black circles) of KCl. The growth curves shown are the result of three independent experiments that gave rise to the same results.

The remaining genes clustered in the "Ion transport" class are involved in the uptake of potassium (*TRK1 *and *ARL1*), ammonium (*MEP3*), phosphate (*PHO88*) and iron (*FET3*, *FRE3*, *FIT2*, *FIT3*). Genes involved in the transport of calcium from the Golgi to the Endoplasmic reticulum (*PMR1 *and *SPF1*) were also grouped in this class (Figure 3). The increased susceptibility to acetic acid of Δ*trk1 *and Δ*arl1 *mutants (Figure 1), deficient in K^+ ^uptake [30,31], suggests that K^+ ^availability in the growth medium may affect yeast resistance to this weak acid. To confirm this hypothesis, yeast susceptibility to acetic acid was compared in a K^+^-free mineral growth medium - ammonium derived growth medium (at pH 4.0) - supplemented with K^+ ^concentrations ranging from 1-20 mM (Figure 4). In the absence of acetic acid, no growth was observed in the basal medium without K^+ ^(results not shown), consistent with the fact that this ion is essential for yeast growth, and the lowest K^+ ^concentration used (1 mM) was growth limiting (Figure 4). Cells growing in the presence of increasing K^+ ^concentrations exhibit an increased tolerance to acetic acid, the effect being more evident for the highest concentration used (20 mM) (Figure 4). The supplementation with 50 mM K^+ ^of the solid growth medium MM4 used to screen the disruptome, which contains 1.7 mM, also led to decreased yeast growth inhibition in the presence of acetic acid (results not shown).

Despite the requirement of several genes encoding iron transporters, or iron-siderophores, for maximal cell protection against acetic acid (Figure 1, Figure 3 and Additional file 1, Table S1), the beneficial effect of growth medium supplementation with iron (concentrations ranging from 1-100 μM of FeSO_4_) in alleviating acetic acid toxicity was not confirmed (results not shown). Nevertheless, the total intracellular iron concentration in an unadapted yeast cell population incubated for 30 minutes in the presence of 60 mM acetic acid (at pH 4.0) was 35.5 ± 1.8 ppm/10^8 ^cells, which is approximately 2-fold higher than the one registered in unstressed cells (20.1 ± 4.2 ppm/10^8 ^cells).

#### Genes involved in carbohydrate metabolism and cell wall structure play a role in yeast tolerance to acetic acid

A high percentage of genes implicated in yeast tolerance to acetic acid play a role in carbon metabolism (Figure 2 and Additional file 1, Table S1). These genes encode proteins involved in glycolysis (*HXK2, PFK1, GCR2, TYE7, GCR1, TYE7*), in the Krebs cycle (*FUM1, KGD2, LPD1, PYC1, PYC2*), in the pentose phosphate pathway (*ZWF1, RPE1*) and in the catabolism and synthesis of reserve carbohydrates (*GPH1, PHO85, PCL7*)(Additional file 1, Table S1 and Additional file 2, Figure S1). Genes encoding a number of components of the respiratory chain (*ATP1, ATP4, ATP5, ATP14, ATP11, COX9, COX11, COX12, COX23, QCR6, QCR7, QCR8, NDE1, COQ5*) and mitochondrial ribosomal proteins (*MRP7, MRPL6, MRPL8, MRPL9, MRPL13, MRPL22, MRPL33, MRPL35, MRPL36, MRPL40, RML2, RSM18, RSM23, MRP16, MRP51, SAM23, POR1, MDM32*) were also found to provide protection against acetic acid (Additional file 1, Table S1 and Additional file 2, Figure S1) suggesting that, even in the presence of glucose, mitochondrial function is essential for tolerance to acetic acid. The transcription factors Rtg1p and Rtg3p, which mediate the nucleus-to-mitochondria signalling pathway, were also identified as determinants of resistance to acetic acid (Additional file 1, Table S1).

Other determinants of resistance to acetic acid clustered in the "Carbohydrate metabolism" class have a function related with the synthesis of β-1,3 glucan (*FKS1, ROM2, ROT2, BEM4*), β-1,6-glucan (*KRE1, KRE6*) and chitin (*CHS1, CHS5*), three cell wall polysaccharides (Additional file 1, Table S1). Other genes related to cell wall function were also identified as determinants of resistance to acetic acid including genes involved in the assembly and remodelling of cell wall structure (*BPH1*, *GAS1, CWH43*) and proteins of the mannosyl polymerase complex II, which promotes the mannosylation of proteins to be incorporated in the mannan layer (*MNN2, MNN9, MNN11, ANP1, KTR4, PMT1, GNT1, GON7, ALG2*) (Additional file 1, Table S1).

#### Genes involved in the uptake and metabolism of amino acids are required for maximal tolerance to acetic acid

A number of genes related to sensing, signalling and uptake of amino acids were also identified as determinants of resistance to acetic acid, including genes involved in intracellular trafficking of the general amino acid permease Gap1p (*GTR1*, *SLM4*, *LTV1*, *RVS161*, *END3*, *UBC4 *and *BUL1*), in the transcriptional control of the yeast response to amino acid starvation (*STP1*) and in the biosynthesis of cysteine and methionine (*CYS3, MET4*), histidine (*HIS4*), glycine (*GLY1*) and glutamate (*GDH1*) (Additional file 1, Table S1 and Additional file 2, Figure S1). The expression of *AGP2 *gene, encoding a low affinity amino acid permease which is transcriptionally regulated by Stp1p, according to the information available in the YEASTRACT database (http://www.yeastract.com)[32], was also found to increase yeast protection against acetic acid (Additional file 1, Table S1). The requirement of genes involved in the biosynthesis of cysteine, glutamate, methionine, histidine and glycine is consistent with the reported decreased concentration of these amino acids inside acetic acid-challenged cells [33]. Since the yeast strain used to carry out this disruptome screening is auxotrophic for histidine and methionine, the supplementation of the MM4 growth medium with glutamate, cysteine and glycine (20 mg/L for each amino acid) was tested and a slight increase in yeast resistance to acetic acid was registered (results not shown).

#### Acetic acid leads to the activation of the Snf1p signalling pathway but, apparently, glucose uptake is not inhibited by acetic acid

**Figure 5:**
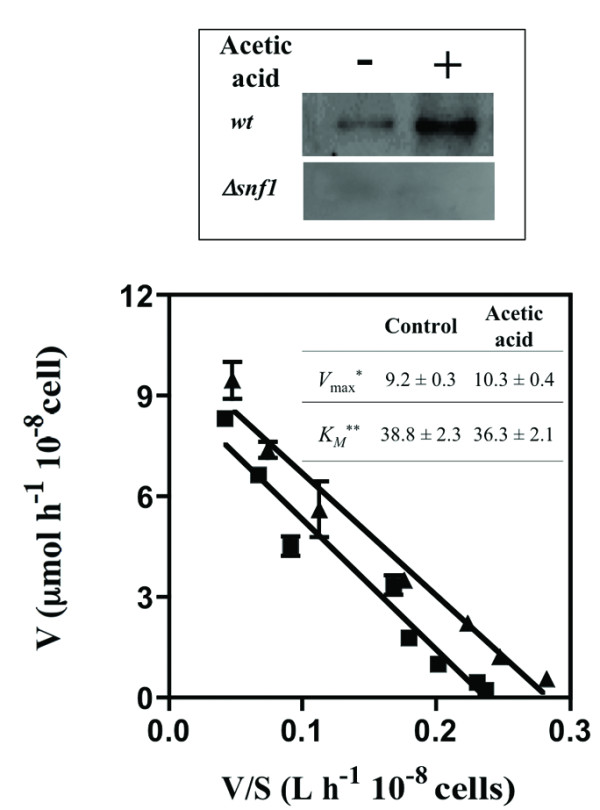
**Acetic acid stress leads to the activation of Snf1p kinase but does not appear to inhibit glucose uptake**. Comparison of Snf1p activity (upper panel) and of glucose uptake rates (lower panel) in yeast cells of the parental strain BY4741 cultivated for 30 minutes in MM4 growth medium (at pH 4.0) (black squares) or cultivated in this same basal growth medium supplemented with 60 mM acetic (black triangles). The determination of Snf1p activity was performed by immunoblotting and was based on the relative quantification of Snf1p phosphorylation at Thr120 residue, as described in Materials and Methods. As a negative control, a similar immunoblot analysis was carried out using protein extracts of the Δ*snf1 *deletion mutant. To estimate the kinetic parameters *K*_*M *_and *V*_max _shown in the insert Table the experimental values of glucose uptake rates were fitted into an Eadie/Hofstee plots. The data presented are medium values from three independent experiments.* μmol per hour per 10^8 ^cells; ** mmol per liter.

A number of mutants susceptible to acetic acid are deleted for genes involved in glucose sensing and signalling, including mutants devoid of genes belonging to several important signalling pathways: the Snf1p-pathway (*SNF1*, *SNF4*, *SNF6*, *MIG1*, *NRG1*), the Ras cAMP/Protein kinase A-pathway (*PDE2*, *RAS2*, *TPK2*, *IRA2*) and the Fermentable Growth Medium- (FGM) signalling pathways (*RIM15*, *SCH9*) (Additional file 1, Table S1 and Figure 1). Evidences suggesting the activation of the Snf1p pathway under acetic acid stress were obtained in this study as described before to occur as part of yeast response to oxidative stress, osmotic shock and heat stress [34]. Indeed, a higher Snf1p phosphorylation level was registered in cells incubated for 30 minutes with 60 mM acetic acid (at pH 4.0), compared to control cells (Figure 5).

Given that the activation of the Snf1p pathway occurs in response to glucose starvation [35] it was hypothesized that acetic acid could have a deleterious effect over glucose uptake into the cell. However, when the initial uptake rate of D-[^14^C]-glucose was compared in cells grown in MM4 growth medium (with 2% glucose) immediately following the addition of acetic acid (60 mM; at pH 4.0), no significant differences in the sugar uptake rates were observed (results not shown). When the cells were pre-incubated with the same concentration of acetic acid used for the transport assays (60 mM) for 5 min in TM buffer (at pH 4.0), the initial uptake rate of glucose decreased, compared to the control cells incubated in the unsupplemented buffer; but this inhibitory effect decrease was not dependent on the concentration of acetic acid used in the pre-incubation step (results not shown). Such effect was considered an artefact attributed to the inactivation of HXT permeases when acetic acid is present as the only carbon source. Given this, the effect of acetic acid in glucose transport capacity was tested using another experimental strategy, using cells cultivated under conditions identical to those used to assess Snf1p activity, that is, after 30 minutes of cultivation in MM4 growth medium either or not supplemented with acetic acid (60 mM, at pH 4.0) (Figure 5). In these transport assays, acetic acid was not added to the assay mixture to avoid the above-referred artifact and also because the concentration of yeast cells necessary to carry this assay (cell suspension with an OD_600 nm _of 50) is much higher than the concentration of the cell suspension in the growth medium (OD_600 nm _of 0.2). This fact prevents the accurate mimicking in the glucose transport assays of the stressing conditions induced by acetic acid in the cultivation medium. The comparison of the kinetic parameters of the glucose transport system indicates an identical affinity for glucose in yeast cells cultivated in the presence or absence of acetic acid (Figure 5) whereas the yeast cells incubated with the acid exhibited a slightly higher maximum glucose uptake rate (Figure 5). This slight increase in glucose transport capacity registered in acetic acid-stressed cells might be related with the up-regulation of the *HXT3 *gene, encoding a low affinity glucose transporter, in these same cells, as suggested by a previous microarray analysis [20]. Altogether, these results indicate that Snf1p is activated in response to acetic acid stress but this adaptive response is, apparently, not caused by the acid-induced inhibition of glucose uptake.

## Discussion

Only 84 of the determinants of resistance to acetic acid identified in our study coincide with those considered as required for yeast resistance to multiple chemical stresses [25] suggesting that a large number of the acetic acid-resistance genes herein identified may play a role in acetic acid tolerance that goes beyond a general contribution to cell fitness under stress. Among the determinants of tolerance to acetic acid that have emerged from our screening we found several genes implicated in the homeostasis and uptake of glucose, potassium, iron and amino acids. This observation appeared to suggest that acetic acid-challenged cells might be starved for these nutrients and that the expression of maximal tolerance to acetic acid would be dependent on cell capacity to efficiently promote their uptake or biosynthesis. Acetic acid, as other weak acids, is thought to dissipate the plasma membrane potential [14,15] affecting secondary active transport. High concentrations of acetic acid have a pro-oxidant action in yeast cells [26,36,37], which may lead to the oxy-radical mediated lipid peroxidation [38] and to the inhibition of the function of membrane-embedded nutrient sensors and transporters. Several genes found to exert protection against acetic acid are involved in the biosynthesis of plasma membrane lipids, including ergosterol (*ERG28*, *ERG4*, *ERG3*, *ERG2*), phospholipids (*SUR4*, *CHO2*, *ARV1*) and sphingolipids (*SUR1*, *SCS7*), which are essential structural membrane components whose concentration in the plasma membrane is modulated under stress [25,39,40]. Indeed, plasma membrane structure is likely to affect yeast tolerance to acetic acid, as found before for other chemical stresses [25]. Despite all the above referred indications, it was not possible to get evidences supporting the idea that the activity of glucose transporters is affected in cells cultivated in the presence of 60 mM of acetic acid, a concentration that induces a period of growth latency in an unadapted cell population (results not shown). Despite that, the Snf1p pathway, considered to be involved in the control of yeast response to glucose starvation [35], is apparently activated in acetic acid-stressed cells. Recently, Snf1p was also found to be activated in response to several environmental stresses, including alkalinization of the growth medium, high osmotic pressure and oxidative stress [34]. Although the signals underlying the control of Snf1p activation are not yet completely understood [35], this activation is known to depend on a high AMP/ATP ratio [41]. Remarkably, acetic acid induces ATP depletion [12,13] and thereby a high AMP/ATP ratio is expected to occur in acetic acid-challenged cells. The depletion of energy caused by acetic acid was attributed to the inhibition of the activity of metabolic enzymes [12] but the up-regulation of the many energy consuming defence mechanisms in acetic acid-stressed cells, including the activation of the proton pumps PM- and V- ATPases [16], should also contribute to energy depletion. The stimulation of the glycolytic flux and of the activity of the Krebs cycle and oxidative phosphorylation registered under acetic acid stress should also contribute to increased ATP synthesis and thus to the prevention of energy depletion in cells challenged with acetic acid [20,33]. This idea is also supported by results of this chemical genomic screening since several mutants deleted for genes related with these metabolic pathways were found to be susceptible to acetic acid.

Other genes herein implicated in yeast tolerance to acetic acid include those involved in cell wall function and in the uptake of potassium and iron. The cell wall-related genes exerting protection against acetic acid are those encoding proteins involved in protein mannosylation (*MNN2 *and *MNN9*) and in the activity and regulation of glucan synthase (*FKS1, ROM2, ROT2, BEM4*). Consistently, glucan synthase was recently proposed as a biological target of acetic acid in yeast cells [42]. The remodelling of yeast cell wall structure in response to acetic acid or to other weak acids is known to be an essential response to reduce the diffusion rate of the undissociated weak acid forms into the cell interior [42,43]. The results gathered so far in the literature sustain the idea that remodelling of yeast cell wall structure is an important process for the increase in tolerance to different weak acids, although the molecular players governing this adaptive response may differ depending on the weak acid tested [14]. In this context, it would be interesting to assess the role played by the expression of *MNN2*, *MNN9*, *ECM31*, *ECM33*, *ROT2*, *CWH43*, *BEM2 *or *ROM2 *genes in acetic acid-induced alteration of cell wall structure since these genes, although providing protection towards acetic acid, are dispensable for tolerance to propionic acid or sorbic acid [44,45].

A set of mutants deleted for genes encoding proteins related with potassium import (Trk1p, Nha1p, Arl1p) were found to be susceptible to acetic acid, suggesting that the uptake of this ion plays a crucial role in yeast response to acetic acid stress. Acetic acid tolerance was correlated for the first time with the presence of increased concentrations of K^+ ^in the growth medium. K^+ ^uptake has been linked to a number of crucial cellular processes in the yeast cell including the regulation of intracellular pH homeostasis, oxidative phosphorylation and sorting of nutrient transporters to the plasma membrane [46-49]. All these biological processes are required for maximal tolerance to acetic acid as suggested by the results of the chemical genomics screening carried out in this work. The activities of Trk1p and Trk2p, the major K^+ ^influx transporters, are essential to the control of internal pH [50] because H^+ ^extrusion mediated by Pma1p is electrogenic and K^+ ^is the major return current in yeast [49]. The increase in potassium uptake in response to acetic acid stress is expected to compensate the stimulation in the activity of Pma1p occurring in these cells [16], thus keeping the electrical balance across the plasma membrane. A similar adaptive response was also proposed to occur in response to sorbic acid [51]. Potassium supplementation of the solid MM4 growth medium used to carry out the phenotypic screening decreased yeast growth inhibition induced by acetic acid even though the concentration of K^+ ^present in the growth medium, close to 15 mM, was above the capacity of the high affinity potassium transport system in unstressed yeast cells (5 mM) [52]). This result is an important finding of this work as it means that tolerance to acetic acid can be alleviated by the manipulation of growth media composition and indicates that the composition of industrial growth media can be optimized to reduce the deleterious effect of acetic acid.

Several genes encoding iron or iron-siderophore transporters exerted protection towards acetic acid and the concentration of iron inside acetic acid-challenged cells was found to be above the concentration present in unstressed cells. Altogether, these observations suggest a stimulation of iron uptake in response to acetic acid. Iron is an essential trace element for yeast growth and, due to its limited bioavailability, *S. cerevisiae *depends on an efficient regulation of various iron uptake systems, including high and low affinity iron transporters and siderophore-iron permeases [53]. The regulation of iron uptake transport systems is, apparently, independent of the cytosolic iron concentration [54,55] being controlled by the rate of synthesis of iron-sulphur proteins in the mitochondria [54]. Indeed, consistent with the here suggested increase of iron uptake in acetic acid-challenged cells, the content of two iron-sulphur cluster proteins, Leu1p and Ilv5p, required for leucine, isoleucine and valine biosynthesis, is higher in acetic acid-challenged yeast cells, compared with unstressed cells [33]. However, the supplementation of MM4 growth medium with iron (concentrations ranging from 1-100 μM) had no detectable positive effect in alleviating yeast susceptibility to acetic acid. Due to its reactivity, iron may be toxic by inducing oxidative stress [53] and yeast cells rely on a tight regulation of the genes and proteins involved in iron acquisition, metabolism and oxidative stress defenses [53]. This tight regulation might limit the simple manipulation of iron composition in the growth medium.

**Figure 6:**
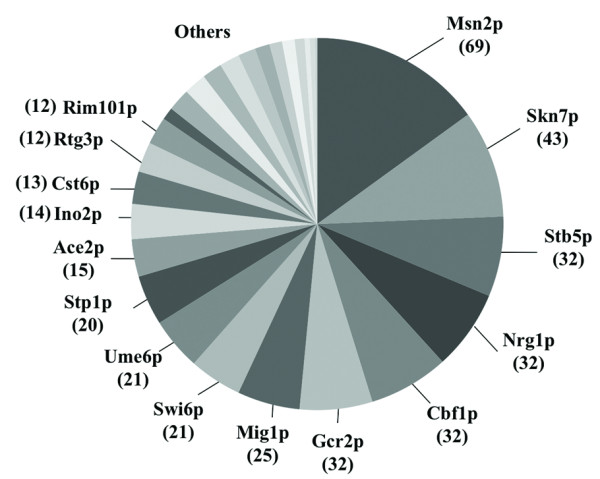
**Clustering of acetic acid-tolerance genes with their corresponding transcriptional regulators that exert protection against the acid**. The yeast genes found to confer protection against acetic acid, according to the results of the disruptome screening, were clustered with their documented regulators using the information available in the YEASTRACT database (September 2010). Only the transcription factors that were also found to exert protection against acetic acid were considered in this analysis. The number of target genes for each transcription factor is indicated in this picture into brackets and the detailed list of genes is provided in Additional file 3, Table S2.

The identification of determinants of yeast tolerance to acetic acid is an essential knowledge to guide the genetic engineering of more robust industrial strains tolerant to this weak acid. In principle all the determinants of resistance to acetic acid identified during this study are candidates for subsequent increased expression in an industrial yeast strain background. Genes encoding transcription factors are, however, of particular interest because their increased expression may result in the simultaneous induction of a set of acetic acid-resistance genes under their control. The manipulation of yeast transcriptional machinery to increase the expression of genes conferring resistance to ethanol and high glucose concentrations was found to be a successful approach to improve the performance of yeast alcoholic fermentations [56]. Our screening uncovered 28 transcription factors required for yeast resistance to acetic acid including Haa1p, Rim101p, Msn2p, whose role in response to acetic acid stress had been described before [14], and others that are here described for the first time (Figure 6 and Additional file 3, Table S2). The functional homologue of Msn2p, Msn4p, also exerts protection against acetic acid [43], although our genome-wide chemogenomics screening failed its identification, possibly because its protective effect is mild, compared to the effect of Msn2p [43]. Using the YEASTRACT database, we searched for the acetic acid-tolerance genes identified in our global phenotypic screening regulated by the transcription factors that also confer maximal protection against acetic acid (Figure 6 and Additional file 3, Table S2). Msn2p and Skn7p, involved in the yeast stress response, and Stb5p, a regulator of multidrug resistance, were found to have the highest percentage of documented targets among the genes required for acetic acid tolerance, thus being interesting candidates for increased overexpression (Figure 6 and Additional file 3, Table S2). Remarkably, the overexpression of the *MSN2 *gene was already found to improve the performance of wine yeast strains leading to higher fermentation productivities [57].

## Materials and methods

### Strains and growth media

The parental strain *Saccharomyces cerevisiae *BY4741 (*MATa, his3Δ1, leu2Δ0, met15Δ0, ura3Δ0*) and the EUROSCARF collection of derived mutant strains, containing all the nonessential open reading frames replaced by the *KanMX *cassette, were used in this study. Cells were batch-cultured at 30°C with orbital agitation (250 rpm) in MM4 liquid medium that contains, per liter: 1.7 g yeast nitrogen base without amino acids or NH_4_^+ ^(Difco Laboratories, Detroit, Mich.), 20 g glucose (Merck), 2.65 g (NH_4_)_2_SO_4 _(Merck), 20 mg methionine, 20 mg histidine, 60 mg leucine, 20 mg uracil, 40 mg tryptophan and 30 mg lysine (all from Sigma; Spain). Yeast Peptone Dextrose (YPD) medium contains, per litter, 20 g glucose, 20 g bactopeptone (Difco) and 10 g yeast extract (Difco). Ammonium phosphate-derived media [58] was used to compare growth of strain BY4741 in the presence of inhibitory concentrations of acetic acid and of increasing concentrations of K^+^. Ammonium phosphate basal medium contains, per liter, a mixture of 0.492 g MgSO_4_.7H_2_O (Merck), 0.02 g anhydrous CaCl_2 _(Panreac), 1.056 g (NH_4_)_2_HPO_4 _(Merck), 3.96 g (NH_4_)_2_SO_4_, 20 g glucose, 2 mg niacin, 2 mg pyridoxine, 2 mg thiamine, 2 mg pantothenate, 0.02 mg biotin [58] and the desired concentration of KCl, in the range of 0-20 mM. For growth of BY4741 strain this growth medium was supplemented with 20 mg histidine, 60 mg leucine, 20 mg, and 20 mg uracil (all from Sigma). The effect in yeast tolerance to acetic acid of MM4 growth medium supplementation with amino acids was compared by adding to the medium 20 mg/L glutamate, 20 mg/L cysteine and 20 mg/L glycine (all from Sigma).

### Screening of the deletion mutant collection for acetic acid susceptibility and data analysis

To screen the Euroscarf deletion mutant collection for sensitivity to acetic acid the strains were grown for 12 hours in MM4 medium in 96-well plates. Using a 96-pin replica platter, the cells were spotted onto the surface of MM4 agarised medium (2% agar). This growth medium, acidified with HCl until pH 4.5, was either or not supplemented with acetic acid at final concentrations of 70, 90 or 110 mM. The stock solution of acetic acid used (5 M) was prepared in water and the pH of this solution was adjusted to 4.5 with NaOH thus increasing the concentration of sodium that is present in the MM4 growth medium (1.7 mM) to a maximum of 3.9 mM in the plates containing 110 mM acetic acid. Depending on the severity of growth inhibition, the plates were incubated at 30°C for 2 or 3 days. Only the mutants that exhibited a cell growth in agar plates not supplemented with acetic acid similar to the parental strain were considered for the identification of acetic acid-susceptibility phenotypes. The mutants whose growth in control agar plates was found to be slightly reduced compared to wild-type strain growth are highlighted in Additional file 1, Table S1. The eventual over- or under- representation of specific terms related with the physiological function of the genes found to be required for acetic acid tolerance was determined using the MIPS functional catalogue(http://mips.helmholtz-muenchen.de/proj/funcatDB/search_main_frame.html). A Fischer exact test was used to correct the data and the enrichment of a functional class was considered whenever the attributed *p*-value is below 0.01. The description of gene function was complemented using the information available in SGD (http://www.yeastgenome.org) and the protein interaction networks were prepared using the STRING software (http://string.embl.de/).

### Assessment of total intracellular iron concentration

Atomic absorption spectroscopy was used to determine the total intracellular iron concentration in cells of *S. cerevisae *BY4741 cultivated in MM4 growth medium (at pH 4.0) or in this same medium supplemented with 60 mM acetic acid. These experiments were carried out in a growth medium at pH 4.0, a pH that is below the one used to carry out the disruptome screening (pH 4.5). Consequently, a lower concentration of acetic acid was used in liquid medium to achieve a similar growth inhibition (60 mM instead of concentrations in the range of 70-110 mM used for the disruptome screening). Due to agarised medium liquefaction as the result of autoclaving, solid medium pH could not be decreased below 4.5. The cells were incubated for 30 minutes in the presence or absence of acetic acid and then harvested by filtration, washed three times with 20 mL EDTA 1 μM (pH 8.0), two times with 15 mL of ice-cold distilled water and left for 12 hours at 65°C in 2 mL 50% (v/v) nitric acid (HNO_3_) for acid hydrolysis [59]. The resulting suspension was centrifuged at 14000 rpm for 5 minutes and the supernatant was recovered to a new tube. Iron quantification by atomic absorption spectroscopy was performed by Laboratório de Análise de Águas of Instituto Superior Técnico (Lisbon, Portugal).

### Estimation of Snf1p activity in cells incubated in the presence or absence of acetic acid stress

The activity of Snf1p was estimated based on the relative quantification of phosphorylation at Thr120 residue, using immunoblotting [34]. Total cytosolic protein extracts (20 μg of protein) of wild-type or ∆*snf1 *cells cultivated for 30 minutes in MM4 growth medium (at pH 4.0), either or not supplemented with acetic acid (60 mM), were separated on 10% acrylamide gels. Subsequent quantitative immunoblotting was performed using an anti-phopho-Thr120-AMPK antibody (Santa Cruz Biotechnology, Germany) and the resulting signals were visualized by chemiluminescence using the ECL-Plus kit (General Healthcare).

### Glucose transport assays

Glucose uptake rates were compared in *S. cerevisiae *BY4741 cells (cell suspension with an OD_600 nm _of 0.2 ± 0.05) cultivated for 30 minutes in MM4 growth medium either supplemented or not with acetic acid (60 mM, at pH 4.0). Cells were harvested, washed with 10 mL ice-cold water and resuspended in TM buffer (0.1 M MES, 41 mM Tris, pH 4.0) to a density of 10^9 ^cells mL^-1^. 40 μL aliquots of these cellular suspensions were transferred to 5-mL Rohren tubes and incubated at 30°C for temperature equilibration. After this period, 10 μL of radiolabeled [^14^C]-glucose (PerkinElmer, MA, USA, 300 mCi mmol^-1^, 11.1 GBq mmol^-1^) was added to each tube by vigorous vortexing. The final concentration of radiolabeled [^14^C]-glucose in the tubes was 200, 100, 50, 20, 10, 5, 2 and 1 mM. These radiolabeled glucose solutions were prepared by dilution of a 1 M radiolabeled [^14^C]-glucose glucose solution. After 5 seconds of incubation of the cells with the radiolabeled glucose, reactions were stopped by vigorous quenching with 3.5 mL ice-cold demineralized water. Cells were subsequently collected by filtration (Whatman GF/C glass microfiber membranes) and the filters were transferred to scintillation vials containing 7 mL liquid scintillation cocktail Ultima Gold MV (Perkin-Elmer). Sugar uptake rates were acquired in duplicates for each sugar concentration and the values obtained were fitted to Eadie-Hofstee plots, using computational assisted linear regression (GraphPad Prism 4.0), to estimate the kinetic parameters *K*_M _and *V*_max_. The glucose uptake rates were also compared in unstressed yeast cells incubated for 5 minutes in TM buffer supplemented or not with acetic acid (final concentrations of 60 or 90 mM) using basically the same transport assay.

## Competing interests

The authors declare that they have no competing interests.

## Authors' contributions

NPM participated in the design and optimization of the disruptome screening experiments, carried out part of the medium supplementation experiments and drafted the manuscript. MP performed the disruptome screening, the glucose uptake assays and contributed to the manuscript draft. JG performed the experiments to test the effect of K^+ ^supplementation in acetic acid-induced yeast growth inhibition. ISC conceived and coordinated the study and participated in the writing of the manuscript. All authors read and approved the final manuscript.

## Supplementary Material

Additional file 1 **Table S1. Yeast genes required for maximal tolerance to acetic acid**. The *S. cerevisiae *BY4741 mutants of the Euroscarf collection was screened to search for the yeast genes conferring protection against acetic acid (70 and 90 mM; at pH 4.5) and those found to be more susceptible to the acid than the parental strain were selected and are listed. Two levels of susceptibility to acetic acid were considered: ++ - highly susceptible mutant; the mutant cells don't grow in the presence of both 70 and 90 mM acetic acid; + - susceptible mutant; the mutant cells exhibit do not grow in the presence of 90 mM and exhibit a reduced growth in the presence of 70 mM of acetic acid when compared to the parental strain. Click here for file

Additional file 2 **Figure S1. Comparison of the susceptibility to acetic acid of a set of deletion mutants tested during the yeast disruptome screening**. Cell suspensions of the parental strain BY4741 or of the indicated deletion mutants were cultivated until mid-exponential phase in MM4 growth medium (at pH 4.5) and then inoculated in plates of this same basal growth medium either or not supplemented with acetic acid (70 and 90 mM; at pH 4.5), as described in materials and methods. Click here for file

Additional file 3 **Table S2. Percentage of "acetic acid-resistance genes" regulated by transcription factors required for maximal tolerance to acetic acid**. The genes found to be required for maximal tolerance to acetic acid were clustered with the transcription factors that were here identified as determinants of resistance to this weak acid, based on the information deposited in the YEASTRACT database (June 2010). Click here for file
