# Supplementary material for: Genome-wide identification of Saccharomyces cerevisiae genes required for tolerance to acetic acid
Source: Microb Cell Fact. 2010 Oct 25;9:79. doi: 10.1186/1475-2859-9-79 (PMC2972246; doi:10.1186/1475-2859-9-79)
Supplement: Additional file 2 — Figure S1. Comparison of the susceptibility to acetic acid of a set of deletion mutants tested during the yeast disruptome screening. Cell suspensions of the parental strain BY4741 or of the indicated deletion mutants were cultivated until mid-exponential phase in MM4 growth medium (at pH 4.5) and then inoculated in plates of this same basal growth medium either or not supplemented with acetic acid (70 and 90 mM; at pH 4.5), as described in materials and methods. [file 1475-2859-9-79-S2.PPT]

## Slide 1
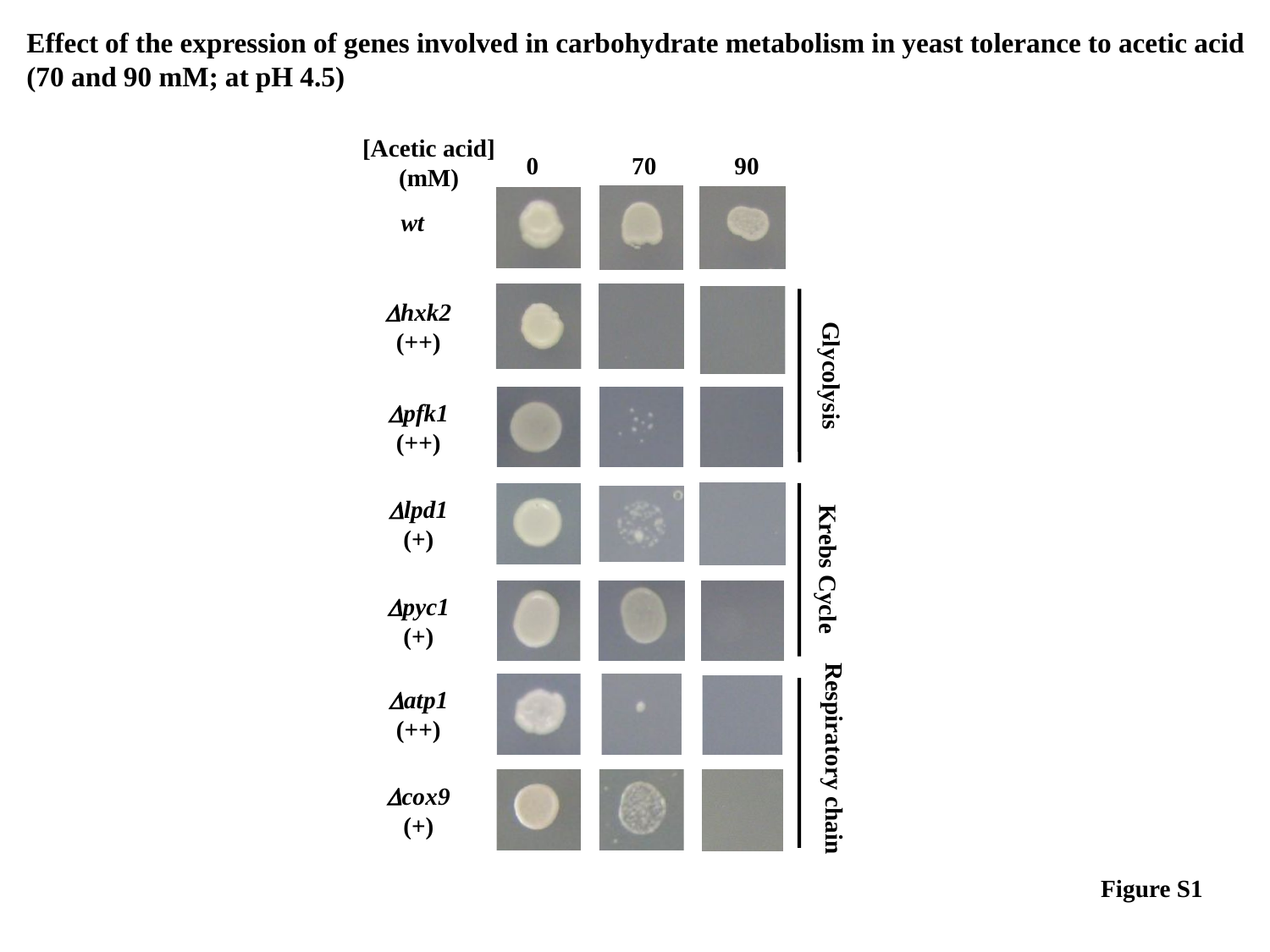

Effect of the expression of genes involved in carbohydrate metabolism in yeast tolerance to acetic acid (70 and 90 mM; at pH 4.5)
[Acetic acid]
(mM)
0
70
90
wt
Δhxk2
(++)
Glycolysis
Δpfk1
(++)
Δlpd1
(+)
Krebs Cycle
Δpyc1
(+)
Respiratory chain
Δatp1
(++)
Δcox9
(+)
Figure S1

## Slide 2
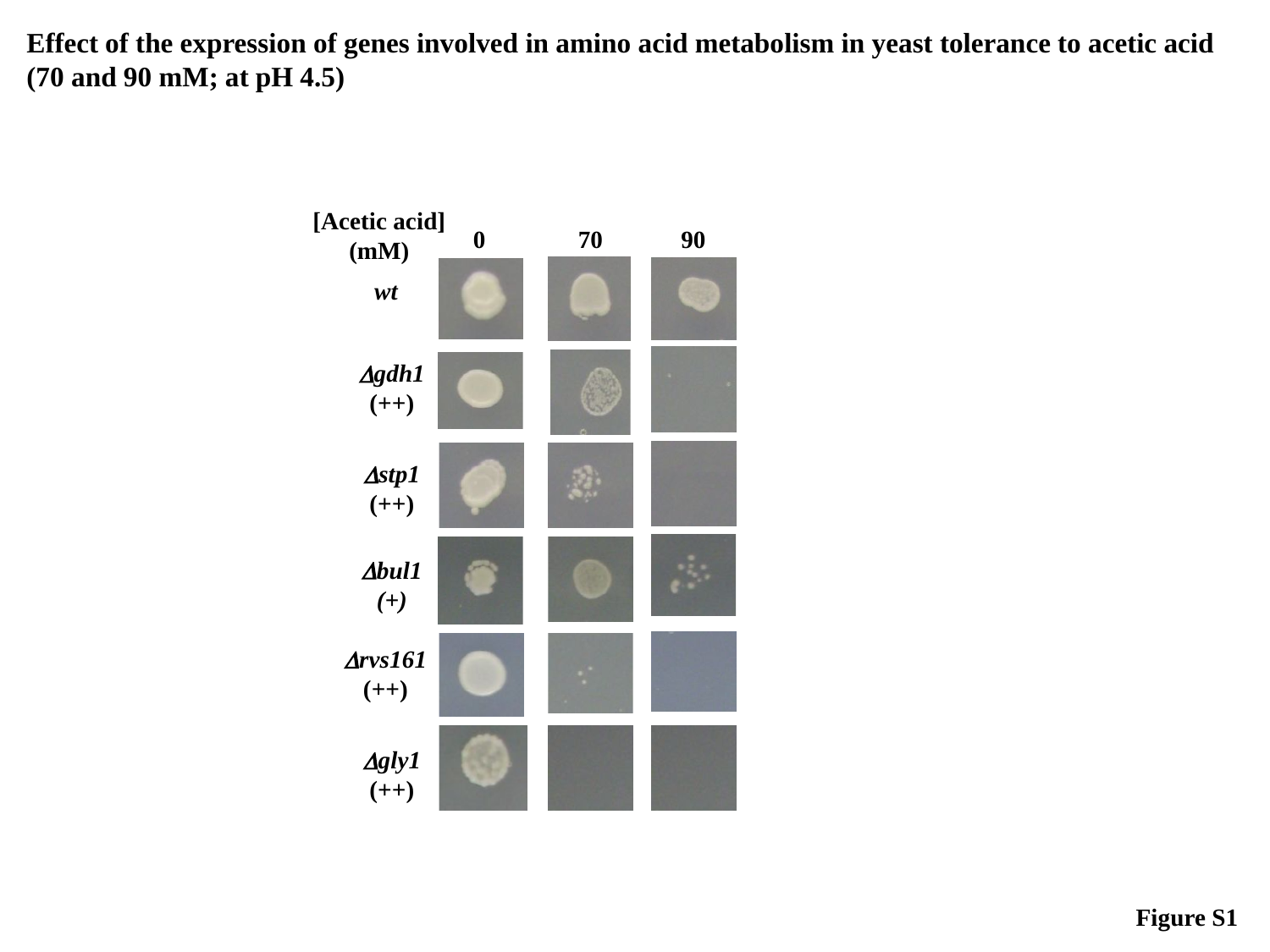

Effect of the expression of genes involved in amino acid metabolism in yeast tolerance to acetic acid (70 and 90 mM; at pH 4.5)
[Acetic acid]
(mM)
0
70
90
wt
Δgdh1
(++)
Δstp1
(++)
Δbul1
(+)
Δrvs161
(++)
Δgly1
(++)
Figure S1
